# Three-dimensional visualization of the cardiac ryanodine receptor clusters and the molecular-scale fraying of dyads

**DOI:** 10.1098/rstb.2021.0316

**Published:** 2022-11-21

**Authors:** Thomas M. D. Sheard, Miriam E. Hurley, Andrew J. Smith, John Colyer, Ed White, Izzy Jayasinghe

**Affiliations:** ^1^ School of Biosciences, Faculty of Science, University of Sheffield, Sheffield S10 2TN, UK; ^2^ School of Biomedical Sciences, Faculty of Biological Sciences, University of Leeds, Leeds LS2 9JT, UK

**Keywords:** dyad, 3D visualization, expansion microscopy, ryanodine receptor, junctophilin, right ventricular failure

## Abstract

Clusters of ryanodine receptor calcium channels (RyRs) form the primary molecular machinery of intracellular calcium signalling in cardiomyocytes. While a range of optical super-resolution microscopy techniques have revealed the nanoscale structure of these clusters, the three-dimensional (3D) nanoscale topologies of the clusters have remained mostly unresolved. In this paper, we demonstrate the exploitation of molecular-scale resolution in enhanced expansion microscopy (EExM) along with various 2D and 3D visualization strategies to observe the topological complexities, geometries and molecular sub-domains within the RyR clusters. Notably, we observed sub-domains containing RyR-binding protein junctophilin-2 (JPH2) occupying the central regions of RyR clusters in the deeper interior of the myocytes (including dyads), while the poles were typically devoid of JPH2, lending to a looser RyR arrangement. By contrast, peripheral RyR clusters exhibited variable co-clustering patterns and ratios between RyR and JPH2. EExM images of dyadic RyR clusters in right ventricular (RV) myocytes isolated from rats with monocrotaline-induced RV failure revealed hallmarks of RyR cluster fragmentation accompanied by breaches in the JPH2 sub-domains. Frayed RyR patterns observed adjacent to these constitute new evidence that the destabilization of the RyR arrays inside the JPH2 sub-domains may seed the primordial foci of dyad remodelling observed in heart failure.

This article is part of the theme issue ‘The cardiomyocyte: new revelations on the interplay between architecture and function in growth, health, and disease’.

## 1. Introduction

Myocardial contraction is enabled through synchronous calcium (Ca^2+^) release within each cardiomyocyte. Type-2 ryanodine receptor (RyR) Ca^2+^ channels clustered on the termini of the sarcoplasmic reticulum (SR) form the primary route of this release of Ca^2+^ into the myoplasm. Spatio-temporal synchrony of the elementary events of Ca^2+^ release (Ca^2+^ sparks [1]) is paramount to achieving the cytoplasmic concentrations required for activating a fast and forceful muscle contraction. Vital to ensuring the regenerative nature and synchrony of Ca^2+^ release are a number of key structural and functional determinants of the local control of RyRs. Structural features enabling this local control include nanoscale co-localization of the RyR clusters with the t-tubules, the L-type Ca^2+^ channel and other components of the excitation–contraction (EC) coupling machinery, the non-uniform post-translational modifications of RyR, and the geometry and distribution of the RyR clusters (see review [2]).

Regulating the organization of RyRs and the EC coupling machinery are several key structural molecules such as junctophilin-2 (JPH2) and amphiphysin-2 (BIN1). In addition to the structural role, JPH2 also directly modulates RyR, physically tethers the SR to the sarcolemma [3], maintains the shape of the t-tubule and regulates the topology of the RyR cluster [4,5]. The loss or downregulation of dyad-related structural proteins has been linked to the aetiology of the maladaptive remodelling of the t-tubules and dyad structures, and concurrently, the dysregulation of the intrinsic Ca^2+^-handling in a range of cardiac pathologies [6]. From super-resolution examination of cardiomyopathies, we [7] and others [8,9] have shown that this remodelling extends to RyR organization at both dyads and sub-sarcolemmal (peripheral) couplons.

Many of the newer insights into sub-cellular remodelling and molecular-scale organization of RyR have come from advanced optical and electron microscopy (EM) techniques. Optical super-resolution microscopy techniques (known best by acronyms such as STED, dSTORM and DNA-PAINT) have led the way in visualizing both cellular compartments and precise molecular targets such as RyRs with nanoscale resolution (less than 250 nm; see review [10]). Tomographic EM techniques have advanced our view of the three-dimensional (3D) complexities of couplon and dyad structures [11–14], while advanced EM-based staining techniques have also enabled *in situ* mapping and counting of macromolecules such as RyR [11]. For example, DNA-PAINT, offering less than or equal to 10 nm resolution and a target-counting algorithm (qPAINT), has become instrumental in quantifying both RyR cluster sizes and the natural heterogeneity in the co-clustering ratio between RyR and JPH2 [15]. The recent application of enhanced expansion microscopy (EExM), a swellable hydrogel approach to obtain resolution of approximately 15 nm, has allowed individual channels to be visualized and counted within clusters located deeper in the myocytes, both in healthy and in failing hearts [7]. However, a major advantage of EExM has been the far superior axial resolution (less than or equal to 35 nm) and imaging depth compared with the molecular-scale imaging protocols such as DNA-PAINT, which currently set the benchmark in optically resolving single dyad targets.

In this paper, we demonstrate the utility of EExM variants (both 10× and 4×; [7]) in visualizing the topologies and geometries of RyR arrays *in situ*. We explore the use of both immunocytochemistry and immunohistochemistry to gain different perspectives of the RyR cluster and t-tubule geometries. By combining 3D visualization with molecular counting of RyR and JPH2, we report the nanoscale dyad remodelling that accompanies the dissipated or fragmented morphology of RyR clusters observed to-date in the cardiac pathology of monocrotaline (MCT)-induced right ventricular (RV) heart failure.

## 2. Methods

### (a) Animal model

Experiments were performed according to the UK Animals (Scientific Procedures) Act of 1986 and with UK Home Office approval and local ethical approval. Animals were housed at 20–22°C, 50% humidity, in a 12 h light/dark cycle and were given ad libitum access to food and water. Adult male Wistar rats weighing 200 ± 20 g were given a single intraperitoneal injection of 60 mg kg^−1^ (of body weight) crotaline (Sigma-Aldrich) (dissolved in 1 M HCl and 140 mM NaCl, pH 7.4 with NaOH) to induce pulmonary arterial hypertension, as detailed previously [16]. Control animals were injected with an equivalent volume of saline solution (140 mM NaCl). Animals were weighed three times weekly for the first three weeks, then daily near the heart failure period (between days 21 and 28). When signs of heart failure were observed (piloerection, cold extremities, lethargy, dyspnoea, 2 consecutive days of weight loss or a 10 g weight loss in a single day [16]), animals were euthanized. Control animals were taken on the median survival day of failing animals. Rats were euthanized by cervical dislocation following concussion in accordance with UK Home Office regulations and local ethical approval. Compared with anaesthesia, this technique does not compromise the heart by exposing it to anaesthetic agents or extended ischaemia.

### (b) Cardiomyocyte isolation and immunocytochemistry

Cardiomyocytes were enzymatically isolated and fixed with 2% paraformaldehyde (Sigma-Aldrich) (w/v) according to a protocol detailed previously [7]. Fixed cells were permeabilized with 0.1% Triton X-100 (Sigma-Aldrich) in phosphate-buffered saline (PBS) for 10 min and then blocked with 10% filtered normal goat serum (NGS, Thermo Fisher Scientific) in PBS for 1 h. Samples were incubated with primary antibodies (type-2 RyR: MA3-916; JPH2: 40-5300, Thermo Fisher Scientific) overnight at 4°C, diluted 1 : 200 and 1 : 250, respectively, in incubation solution containing (w/v or v/v) 0.05% NaN_3_, 2% bovine serum albumin, 2% NGS and 0.05% Triton X-100 dissolved in PBS. Samples were washed in PBS and then incubated with secondary antibodies (anti-mouse Alexa Fluor 488, anti-rabbit Alexa Fluor 594, Thermo Fisher Scientific) for 2 h at room temperature, diluted 1 : 200 in incubation solution. Samples were washed in PBS and imaged to obtain pre-expansion images.

### (c) Cardiac tissue processing and immunohistochemistry

For immunohistochemistry experiments, different hearts were dissected into left and right ventricles and fixed by immersion in 1% paraformaldehyde (w/v) in PBS at 4°C for 1 h. Fixed tissue was then washed and cryoprotected by immersing it through a series of sucrose solutions (series of 10, 20 and 30% w/v). Excess sucrose solution was removed before a thin layer of O.C.T. compound (Tissue-Tek) was applied to coat the tissue. The tissue was snap-frozen for 2 min by immersion in methylbutane (Sigma) within a container of liquid nitrogen. Frozen tissue blocks were cryosectioned with a Feather Blade at −20°C. Ten microlitre-thick sections were obtained and attached to coverslips until immunofluorescent labelling.

Cardiac tissue sections were treated with Image-iT FX signal enhancer (Thermo Fisher Scientific) for 1 h at room temperature (20–22°C) prior to incubation with primary antibodies (RyR: MA3-916 (Thermo Fisher Scientific); sodium–calcium exchanger: R3F1 (Swant); caveolin-3: 610420 (BD Transduction)), overnight at 4°C, and diluted 1 : 200 in incubation solution. After washing in PBS, sections were then incubated with secondary antibodies (as above) for 2 h at room temperature, in incubation solution.

### (d) Expansion microscopy

Immunolabelled samples were incubated with 0.1 mg ml^−1^ acryloyl-X (Thermo Fisher Scientific) in PBS overnight at 4°C, then washed in PBS immediately prior to addition of gel solution.

10× EExM was performed on isolated cardiomyocytes as detailed previously [7,17]. X10 gels (4 : 1 molar ratio of dimethylacrylamide (Sigma-Aldrich) and sodium acrylate (Sigma-Aldrich), dissolved in deionized H_2_0 (dH_2_O)) were made according to the previous recipe [18,19]. Gel solution was made fresh and bubbled with nitrogen gas for 1 h on ice. Potassium persulfate (Sigma-Aldrich) was added from a fresh 0.036 g ml^−1^ stock to 0.4% molar relative to the monomer concentration, and the solution was bubbled for another 15 min on ice. Five hundred microlitres of the gel solution was mixed rapidly with 2 µl of *N*,*N*,*N*′,*N*'-tetramethylethylenediamine (Sigma-Aldrich) and quickly added to the sample coverslip. The polymerization chamber, comprising the sample coverslip with two coverslip spacers (one each side), was sealed with a top coverslip. Gels were polymerized after 2 h. The major axes of the gel were measured to calculate the pre-expansion size.

4× EExM was performed on tissue sections, similar to that described for isolated cardiomyocytes previously [17]. The gel solution, prepared according to the proExM protocol previously described [20], was made in advance and defrosted from frozen aliquots. Tissue sections were incubated with monomer solution containing (w/v, Sigma-Aldrich) 8.6% sodium acrylate, 2.5% acrylamide, 0.15% *N*,*N*'-methylenebisacrylamide, 11.7% NaCl, PBS, 0.1% ammonium persulfate and 0.1% *N*,*N*,*N*′,*N*'-tetramethylethylenediamine first for 30 min at 4°C and then for 2 h at 37°C.

Polymerized gels were removed from the coverslip chamber and placed into six-well plates to undergo digestion in 0.2 mg ml^−1^ proteinase K (New England Biolabs) dissolved in digestion buffer (50 mM Tris pH 8.0 (Thermo Fisher Scientific), 1 mM ethylenediaminetetraacetic acid (Sigma-Aldrich), 0.5% Triton X-100, 0.8 M guanidine HCl (Sigma-Aldrich) and dH_2_0) overnight at room temperature. Gels were expanded by shaking in dH_2_O until the gel expansion reached a plateau, replacing the dH_2_O every hour. The final gel size was measured to calculate the macroscale expansion factor, in relation to the pre-expansion size.

### (e) Image acquisition

Expanded gels were placed into acrylic chambers with a square cut-out, attached to a no. 1.5 glass coverslip (Menzel Gläser), which had been coated with 0.1% (v/v) poly-l-lysine (Sigma-Aldrich) at room temperature for 30 min. Airyscan imaging was performed on an inverted LSM880 (Carl Zeiss, Jena), with a Plan-Apochromat ×63 1.4 NA objective with a working distance of 0.19 mm. AlexaFluor 488 and AlexaFluor 594 were excited with 488 and 561 nm DPSS lasers, while emission bands were selected using the in-built spectral detector.

### (f) Image analysis

All 3D visualizations including 3D isosurface and volume rendering were performed in ParaView (Kitware, Los Alamos). Applications of colourtables, two-channel overlays and maximum-intensity projections were performed in FIJI (ImageJ 1.53c) with the BioFormats plugins.

The post-acquisition image processing and the measurements on RyR and JPH2 clusters are detailed in electronic supplementary material, Methods.

All statistical tests on measurements reported in this paper were non-parametric and were performed in GraphPad Prism.

## 3. Results

### (a) Characterization of 3D resolution attainable with enhanced expansion microscopy

**Figure 1.**
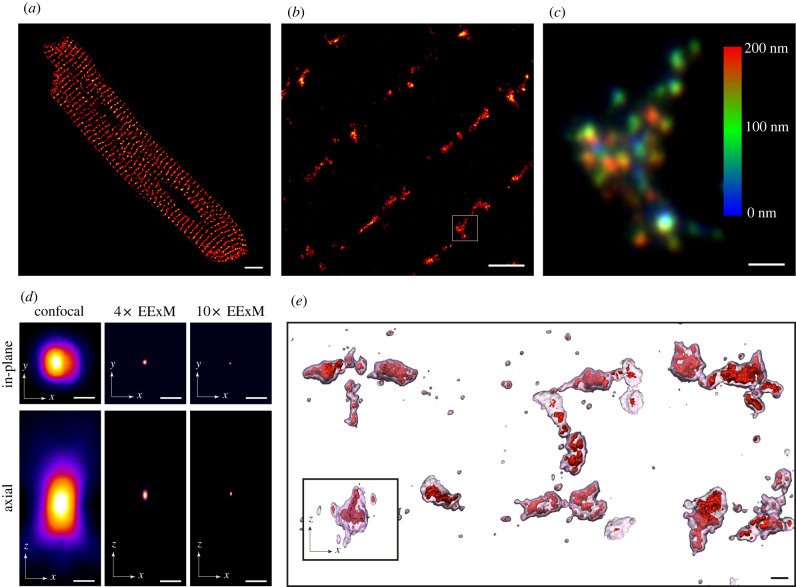
3D complexity of ryanodine receptor clusters visualized with EExM. (*a*) A typical 2D Airyscan image of RyR immunofluorescence labelling in a rat ventricular myocyte. (*b*) A 2D Airyscan image of a region in a similar cell, acquired with the 10× EExM protocol. (*c*) Magnified and projected view of the region of interest in (*b*), colour-coded for axial depth. Colour bar indicates depth in nanometres. (*d*) To-scale comparison of the effective point spread functions of confocal, 4× EExM and 10× EExM, shown in the in-plane (upper) and axial dimensions (lower). (*e*) Isosurface-rendered 3D reconstruction of two adjacent rows of RyR, visualized with 10× EExM (red) and a resolution equivalent to 4× EExM (transparent pink), illustrating the superior resolution and better-resolved curvatures of the RyR clusters with the former; inset shows the *x–z* view of an exemplary cluster whose curved topology in the *z*-dimension (dashed line) was better resolved with 10× EExM than with 4× EExM. Scale bars: (*a*) 5 µm; (*b*) 1 µm; (*c*,*e*) 100 nm, (*d*) 250 nm.

10× EExM is an imaging protocol that combines the approximately 1000-fold volume inflation of a hydrogel-based fluorescent imprint of the sample, based on a protocol called ‘X10 ExM’ [18], with a further twofold resolution improvement afforded by Airyscan compared with regular confocal microscopy [7]. Figure 1*a* illustrates an exemplary confocal micrograph of immunofluorescence labelling of RyR in a rat ventricular myocyte. With 10× EExM, not only can the banded RyR staining morphology be resolved to be domains with intricate and varied shapes (figure 1*b*), but also the nanoscale punctate labelling densities, which represent individual RyRs [15], are clearly observable. Maximum-intensity *z*-projection of one of these clusters, colour-coded for depth (figure 1*c*), reveals the intrinsic ability of 10× EExM to resolve the positions of individual RyRs both in-plane and axially.

This improvement in both in-plane and axial resolution was a direct result of the effective down-scaling of the confocal point-spread function by a factor of approximately 20 in each of the three dimensions (figure 1*d*). Even 4× EExM (which employs the widely used proExM protocol [20]) offered an eightfold resolution improvement over the confocal point spread function (see simulations in the electronic supplementary material and fig. S5 of [7]). When imaging the 3D topologies of the RyR clusters, 4× EExM therefore produced sharply defined cluster shapes (transparent pink isosurface in figure 1*e*). With 10× EExM, however, we were able to observe the cluster sub-structures, including curved or folded arrays of RyR (solid red isosurface in figure 1*e*), which were not observed in previous super-resolution studies of RyRs deeper in the cell interior [9,21,22]. With an axial resolution of approximately 35 nm, 10× EExM was also capable of resolving curvatures of the RyR clusters that extended in the *z*-dimension (inset of figure 1*e*).

### (b) Visualizing transverse and longitudinal perspectives of dyads

**Figure 2.**
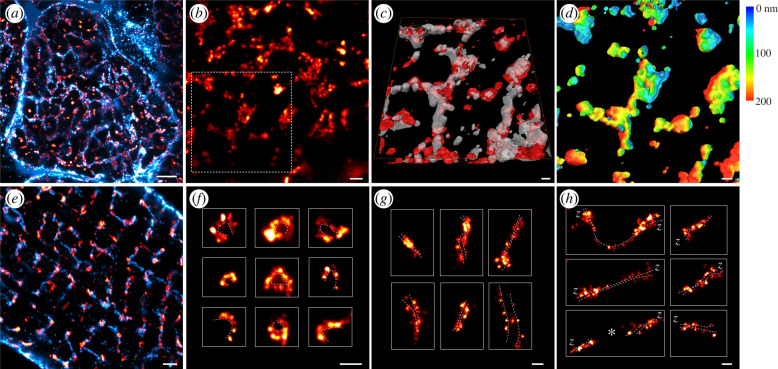
Transverse and longitudinal views of the organization and geometries of RyR clusters. (*a*) Transverse view of a ventricular myocyte within a cryosection of RV tissue, labelled for RyR (red) and t-tubules (cyan) and imaged with 4× EExM, illustrated by a maximal intensity projection across a 1 µm-deep volume. (*b*) RyR labelling in a 2D, transverse view shows a highly punctate morphology. (*c*) Isosurface rendering of the RyR (red) and t-tubule (cyan) channels, magnified from the region of interest in (*b*), illustrates that when imaged in transverse view, even 4× EExM can reveal the topological complexities of the RyR clusters. (*d*) These topologies are shown more directly with an axial depth-encoded (scale shown in nanometres) colouring of a smoothed isosurface rendering of the RyR volume. (*e*) 10× EExM of enzymically isolated myocytes is more suitable for visualizing RyR (red) and t-tubules (cyan) throughout the cell interior in longitudinal view. In this view, we can identify RyR clusters arranged in varying orientations relative to the image plane: (*f*) visualized in ‘end-on’ orientation; (*g*) clusters located within the transverse bands of RyR, visualized in ‘side-on’ orientation; and (*h*) clusters observed to be extending longitudinally between transverse bands, visualized similarly in ‘side-on’ orientation. Asterisk indicates an example of consecutive longitudinal clusters located on a single longitudinal tubule. The dashed lines approximate the t-tubule location and geometry (*f*–*h*), which are *inferred* based on the orientation and geometry of the RyR labelling. Scale bars: (*a*,*e*) 1 µm; (*b*,*f*–*h*) 250 nm; (*c*,*d*) 100 nm.

We and others have previously shown that re-orienting myocytes to scan the RyR clusters in transverse view allows a more complete and spatially resolved visualization of the network structure of the dyads and t-tubules compared with the conventional approach (imaging myocytes in longitudinal orientation; [23,24]). By combining 4× EExM with tissue immunohistochemistry, we examined myocytes that were physically sectioned in the transverse plane and visualized these structures at an in-plane resolution of less than 40 nm and an axial resolution of less than 90 nm (figure 2*a*). Examination of a zoomed-in region of the 4× EExM image of RyR showed clusters similar in outline to previous 2D and 3D dSTORM images [21,25]. The sub-structures of the clusters were heterogeneous and punctate (figure 2*b*). This view was ideal for examining the network of t-tubules (visualized with a combined immunostain of caveolin-3 and the sodium–calcium exchanger, as described previously [26]) which extend across the cell's *z*-line and optically resolve the tessellation of individual RyR clusters around the tubules. 3D isosurface rendering of the boxed region in figure 2*b* is shown in figure 2*c*. t-Tubule labelling in this region illustrates the intricate nanoscale topologies of the t-tubular network (translucent cyan in figure 2*c*), while a lightly smoothed rendering of the RyR labelling (red) illustrates the curvatures of the RyR arrays around the local t-tubule geometry. Depth-encoded coloured isosurface rendering of the smoothed RyR image further illustrates the intricate curvatures of the RyR clusters that were previously not visualized with optical microscopy methods (figure 2*d*).

While 10× EExM is currently not compatible with myocardial cryosections (owing to the need for more thorough digestion enabling increased expansion factor), it is still highly suited for imaging the RyR clusters located deeper in the interior of enzymatically isolated myocytes. Typically, imaging of these samples was performed in longitudinal orientation (figure 2*e*). The various orientations and geometries of RyR clusters were clearly discernible even in 2D Airyscan images. Noteworthy among the clearly identifiable geometries were the end-on view of the dyads where the curvatures of the RyR clusters were visible (figure 2*f*) and the side-on view of clusters located at transverse (figure 2*g*) and longitudinal tubules (figure 2*h*). In the latter views, a characteristic groove in the intensity topography of the RyR cluster allowed us to predict the geometry of the local t-tubule (dashed lines), even when fluorescent markers of the t-tubules were not available.

### (c) Variable junctophilin-2 sub-domains in peripheral and deeper couplons

**Figure 3.**
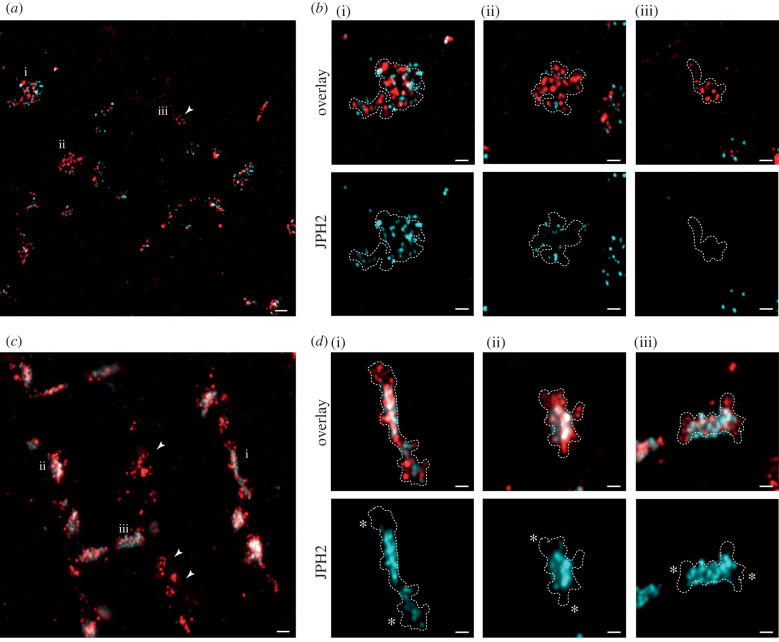
Molecular positioning of RyR and JPH2 near the cell surface and deeper in the cell interior. (*a*) A 10× EExM image taken near the surface of a ventricular myocyte illustrates the organization of RyR clusters (red), co-localizing frequently with punctate JPH2 labelling (cyan), across a 2D geometry of the nanodomain. A small proportion of (typically smaller) RyR clusters that lacked JPH2 were also noted (arrowhead). (*b*) Magnified view of three exemplar clusters (denoted i–iii in (*a*)) illustrating the boundary of the RyR cluster. JPH2 in these clusters shows high variability in both the density and the spatial uniformity inside the RyR cluster boundary. (*c*) A shallow maximum-intensity projection of a 100 nm-deep 10× EExM image volume of RyR (red) and JPH2 (cyan) notably identifies more frequent RyR clusters devoid of co-localizing JPH2 (arrowheads) in the cell interior. (*d*) Magnified views of three exemplar regions show that the RyR cluster boundaries (dashed lines) consistently extend beyond the longitudinal poles of the intrinsic JPH2 sub-domains (asterisks). Scale bars: (*a*,*c*) 200 nm; (*b*,*d*) 100 nm.

10× EExM revealed punctate labelling morphologies of both JPH2 (cyan) and RyR (red) in the periphery of healthy rat ventricular myocytes. These RyR arrays were planar, as reported previously [7], and therefore the mutual arrangement of RyR and JPH2 could be fully appreciated in 2D images similar to figure 3*a*. The vast majority of the RyR clusters co-clustered with JPH2 (despite some exceptions, arrowhead); however, the relative density of JPH2 across these domains varied from cluster to cluster. Figure 3*b* illustrates three exemplary clusters which consist of typically punctate patterns of RyR (dashed lines outline the RyR arrays), but also highly heterogeneous organization of JPH2 within the cluster domain. JPH2 occupied the entire area in some clusters (figure 3*b*(i)) but only small sub-domains in most others (figure 3*b*(ii)).

By comparison, RyR clusters devoid of JPH2 were more frequently observed in the interiors of these myocytes (arrowheads; figure 3*c*). In clusters where RyR and JPH2 were co-localized, JPH2 appeared to be highly enriched in clearly resolvable sub-domains, usually in the central regions of the cluster (see exemplary clusters in figure 3*d*(i–iii)). Coinciding with the lack of JPH2 near the poles of the cluster (asterisks in figure 3*d*), we also observed a broadening of the RyR cluster and a looser organization of the RyRs near the poles.

### (d) Ryanodine receptor cluster fragmentation and molecular-scale fraying in right ventricular failure

**Figure 4.**
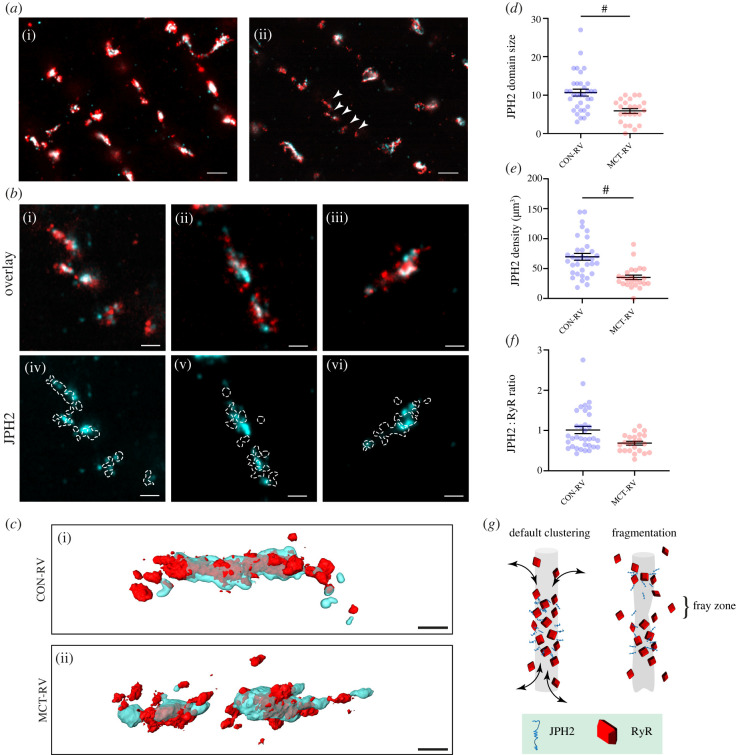
Molecular-scale remodelling of RyR clusters and intrinsic JPH2 organization in RV failure. (*a*) Exemplar 10× EExM images of RyR (red) and JPH2 (cyan) organization in the interiors of myocytes isolated from CON-RV (i) and MCT-RV (ii). Note the shorter RyR and JPH2 domains in the latter compared with the control (arrowheads). (*b*) Magnified view of three exemplar clusters (i–iii) showing a fragmented RyR (red) cluster morphology in MCT-RV overlaid with the local JPH2 sub-domains (cyan); (iv–vi) depict the poor alignment of the punctate RyR densities (i.e. frayed pattern) in relation to the JPH2 sub-domains. (*c*) 3D surface render of exemplar RyR clusters (red) and JPH2 sub-domains (cyan) from CON-RV (i) and MCT-RV (ii) cardiomyocytes. Scale bars: (*a*) 500 nm; (*b*,*c*) 100 nm. Dot plots compare (*d*) the JPH2 domain size (in terms of number of detected JPH2 puncta per domain), (*e*) the density of JPH2 organization within the nanodomain, and (*f*) the estimated JPH2 : RyR ratio between CON-RV and MCT-RV. Mann–Whitney tests (^#^*p* < 0.0001, d.f. = 57 clusters). Whiskers denote mean and s.e. (*g*) Schematic depicting hypothesized sub-domain remodelling in RyR clusters. In the healthy phenotype, JPH2 occupies a central sub-domain of the dyad while the poles, devoid of JPH2, may serve as turnover domains (arrows) where RyR organization may be either looser or physically broader. In maladaptive remodelling, the downregulation of JPH2 may lead to fragmentation of the structural sub-domain along with potential remodelling of the local t-tubule and concurrent fragmentation of the RyR cluster, in an unknown temporal sequence. The fragmentation of the JPH2 sub-domain may cause ‘fray zones’ that function as turnover domains, giving rise to the dissipated or frayed morphology observed in EExM image data.

We used 10× EExM to examine the 3D features of RyR cluster remodelling in RV myocytes isolated from rats with MCT-induced RV failure (MCT-RV). Figures 4*a,b* compare exemplary 10× EExM images of RyR (red) and JPH2 (cyan) in the interiors of control RV (CON-RV) myocytes and MCT-RV myocytes, respectively. At low magnification, both scenarios featured RyR clusters that were predominantly aligned transversely. In both cases, most of the RyRs appeared to co-cluster with JPH2. The cluster fragmentation in MCT-RV cells featured distinct groupings of smaller RyR clusters in 1–2 µm-wide sub-cellular regions, which were otherwise devoid of larger RyR clusters (arrowheads in figure 4*a*(ii)). Closer examination of these regions showed that each cluster fragment still featured smaller sub-domains of JPH2; however, the RyR puncta appeared more scattered *around* the JPH2 sub-domain (figure 4*b*(i–iii)). Examining the RyR position in relation to the JPH2 sub-domains (figure 4*b*(iv–vi)), the RyRs appeared more scattered around the edges of the JPH2 structural domains, resembling a fraying rope (see 3D isosurface visualization in figure 4*c*).

The punctate nature of the JPH2 morphology in 10× EExM images allowed us to perform counts of the labelling units. From this analysis, we observed that there was a reduction in the JPH2 puncta in MCT-RV clusters in comparison with CON-RV (mean 5.88 JPH2s per cluster in MCT-RV versus 10.65 in CON-RV; figure 4*d*). This reduction was also accompanied by an approximately 45% reduction in the mean density of JPH2 within the cluster volume (figure 4*e*). While we did not observe a statistically significant change in the mean ratio of the number of JPH2 puncta to the RyR puncta in a given cluster, we did observe a drop in the variability of this ratio in MCT-RV, compared with CON-RV (s.d. 0.27 in MCT-RV versus 0.53 in CON-RV; figure 4*f*). The shift in JPH2 : RyR ratio happened to a lesser extent for MCT-LV (electronic supplementary material, figure S1).

## 4. Discussion

### (a) Enhanced expansion microscopy as a tool for visualizing topologies and molecular organization of dyads

A handful of optical super-resolution techniques, including DNA-PAINT and EExM [7], currently offer the capability to resolve individual RyRs *in situ*. However, a major shortcoming in the use of localization-microscopy to visualize cardiac dyads has been the limited capacity to resolve 3D complexity of the geometries and the topological features. We previously predicted the possibility of fully resolving individual RyRs within the curvatures of the dyad (see a simulation in the electronic supplementary material and fig. S5 of [7]). Both the 2D and 3D visualizations shown in this paper demonstrate the full range of geometries and morphologies of RyR clusters observed in cardiomyocytes using 10× EE×M. 4× EExM allows visualization of RyR clusters comparable with that achievable with 3D dSTORM. Its compatibility with both isolated myocytes and myocardial cryosections, and capacity for a complete visualization of the t-tubule network along with the tessellation of RyR clusters make 4× EExM an arguably more versatile imaging modality.

The most significant advantage of using EExM for 2D or 3D imaging of cardiac RyR clusters is that it can report the precise boundaries of the cluster, and the likely geometry of the dyad as well as the positions of the RyRs. By comparison, dSTORM (in a typical 2D- or 3D-localization implementation) lacks the localization precision to spatially resolve individual channels *within* a cluster [15]. The advantage in choosing techniques with sub-15 nm in-plane resolution (and ideally similar axial resolution) is the versatility to observe protein cluster features such as topology, perform co-localization measurements and visualize molecular-scale remodelling in 3D. On this principle, more details can be gained by 10× or higher-order ExM implementations, such as adaptations of iterative ExM [27]. However, pragmatically a balance needs to be sought between the resolution gains, and the ease and reliability of the chosen expansion protocol; with the current state of the art, we find that 10× EExM is a convenient protocol that works well with isolated cardiomyocytes.

The isotropy of gel expansion remains a point of concern for many ExM users in the context of artefacts and aberrations that it can introduce to the structures visualized. We have used the sarcomeric length of myocytes expanded within the 10× EExM gels and the width of the *z*-line as intrinsic markers to characterize the isotropy and the linearity of the gel expansion (see electronic supplementary material, figure S2). With free-radical polymerization-based ExM, the distortions introduced from gel anisotropy or deformations tend to be in the larger (micrometre to millimetre) length-scales [28]. The linear error relating to these distortions for the X10 gel recipe used in our experiments (across the 50–500 nm length-scales) is estimated to be approximately 3–5% [18]. Considering the potential distortions introduced to the image by the large probes such as antibodies and fluorophores [29], this error is minor.

### (b) Sub-domains of junctophilin-2 occupancy and model of dyad fraying in pathology

We have observed distinct sub-domains of JPH2 within the dyad, which have not been observed with previous dSTORM imaging owing to insufficient resolution. In sub-sarcolemmal couplons, the organization of JPH2 may be more variable, leading to a structure that is more conducive to the rapid remodelling or dynamic re-organization seen previously [22,30]. In dyads located deeper into the cell interior, JPH2 localizes more consistently to structural sub-domains occupying the central region. The looser co-clustering of RyR with JPH2 at the poles of the dyads may represent turnover domains that could enable exchange of RyRs (similar to turnover of gap junction plaques [31]; also figure 4*g*, left). Such a polarity of RyR organization in the dyads may indicate the local connectivity of the SR/endoplasmic reticulum, which would form trafficking routes and lateral insertion points for dyad proteins, similar to those seen in synaptic proteins [32]. The denser occupation of JPH2 in the central portions of the larger RyR clusters could indicate a lower propensity for remodelling or mobility of these domains.

In MCT-induced RV failure, we have consistently observed RyR cluster remodelling, particularly fragmentation of clusters (see Sheard *et. al.* [7], and electronic supplementary material, figure S3) coinciding with the downregulation of structural proteins such as BIN1 and JPH2 (*via* either microRNA silencing, calpain cleavage or trafficking misdirection) [16,33]. By quantifying JPH2 puncta and labelling density, we have demonstrated that there is JPH2 downregulation inside the MCT-RV dyad geometry (an observation made previously by Western blot [33]). Our spatially resolved analysis shows that the JPH2 : RyR ratio becomes less heterogeneous in MCT-RV. In previous 10× EExM analyses, we showed that the RyR–RyR spacings in the left ventricles of animals with RV failure were relatively unaltered [14]. Our quantification of JPH2 cluster size, intrinsic JPH2 density and the JPH2 : RyR ratio in MCT-LV suggests that JPH2 expression is also largely unaltered compared with the control-LV (or control-RV; electronic supplementary material, figure S1).

The morphology of RyR arrangement in the regions of the clusters lacking JPH2 in MCT-RV myocytes leads us to conclude that JPH2 plays a direct role in the maintenance of the closely packed RyR arrays. The regression of JPH2 sub-domains may therefore cause the fragmented appearance of the RyR cluster (figure 4*g*, right). Given the role of JPH2 in organizing the local t-tubule membranes, the RyR ‘fray zones’ may also represent the primary foci for the t-tubule remodelling observed in cardiac hypertrophy and myopathies. Confirmation of intrinsic t-tubule remodelling would require a triple-colour visualization of RyR, JPH2 and the t-tubules, which we are currently unable to perform owing to lack of compatible and reliable antibody markers.

### (c) Bottlenecks and motivations for expansion microscopy of cardiac ryanodine receptor clusters

EExM, despite the far superior 3D resolution that it offers in resolving the topologies of both the dyads and individual RyRs, still requires a number of calibrations and tests. These include independent estimates of the gel expansion factors and isotropy of expansion (see detailed discussion in [17]). Combining the X10 expansion protocol with imaging modalities such as STED or SIM can further improve the resolution attainable to a sub-10 nm regime. The skill and experience involved in the handling and fluorescence imaging of the hydrogels remain a significant human element in the methodology. However, the emergence of newer gel formulae capable of greater expansion isotropy [34], and pan-stains that can delineate other cellular compartments [35], enables increasingly greater reliability and versatility of ExM for investigations such as this. Despite the unique opportunity to visualize the transverse aspect of the t-tubular network and dyads with 4× EExM combined with immunohistochemistry, we note the excessive rigidity (owing to the extracellular matrix) inherent to myocardial tissue undergoing pathological remodelling. While the size and specificity of the fluorescent probes are critical (the RyR and JPH2 antibodies used here have been extensively characterized previously), it should also be noted that post-labelling and re-embedding ExM gels can further improve the completeness and geometric accuracy of the structures [36].

The far superior 3D information offered by EExM compared with other recent implementations of super-resolution microscopy remains a key reason for its utility. The 3D contextual information it has provided us in terms of t-tubule geometries, organization of structural proteins such as JPH2, and examining RyR cluster fragmentation in heart failure has been pivotal to our observation of JPH2 sub-domains. Having used this method in our recent studies, we also observe the greater accessibility and uptake of the EExM protocols by traditional cell biologists, compared with other super-resolution techniques. We therefore anticipate broad applications and refinement of the method within the cellular cardiac discipline in the next few years.

## 5. Conclusion

We have demonstrated that combining EExM with 3D visualization can reveal the diverse topologies and geometries of RyR clusters in cardiac muscle. Owing to the fine in-plane and axial resolution afforded by 10× EExM, we have been able to identify sub-domains within the cardiac dyad that are occupied by the structural protein JPH2. In RV failure, we observe fragmentation of these JPH2 sub-domains, coinciding with a drop in the intrinsic JPH2 density and the dyad-to-dyad heterogeneity in the ratio between RyR and JPH2. Using 3D visualization of individual RyRs, we identify waning or fragmenting JPH2 sub-domains as the likely sites of dyad remodelling.

## Data Availability

All data included in the paper are processed data. In the electronic supplementary material, data (zip) file [37], we have attached exemplar datasets and exemplar Python and IDL scripts used for the quantitative image analysis.
